# A statement of the rights of scientists and engineers

**DOI:** 10.4103/0971-6203.44471

**Published:** 2008

**Authors:** William Hendee

**Affiliations:** Editor, Medical Physics Chair, Publications Committee, International Organization of Medical Physics, PO Box 170970, Whitefish Bay, WI 53217. E-mail: whendee@mcw.edu

Several months ago, the President of the International Organization of Medical Physics (IOMP) asked me to write a Bill of Rights for Scientists and Engineers. His request subsequently endorsed by the IOMP Council, reflected concerns about the intrusion of political, cultural, and religious pressures on the research of scientists and engineers, and on the dissemination of the results of research to governmental policy-makers and to the public at large. The Bill of Rights reproduced below responds to the IOMP President's request, and has been approved by the IOMP Council and by the Executive Committee of the International Federation of Medical and Biological Engineering. The Bill of Rights is currently under consideration by the International Council for Science.

A few months ago, the IOMP Publications Committee, which I chair, approved an editorial on, “A Concern about Plagiarism” that has been published in many of the world's medical physics journals, including the Journal of Medical Physics. The IOMP Publications Committee intends to disseminate the present editorial in which the Bill of Rights for Scientists and Engineers is embedded, to the same journals for potential publication. The Publications Committee is composed of the editors of all of the world's medical physics journals, so a decision to disseminate the editorial by the Publications Committee is in effect a proposal to the editors to publish the editorial on ‘Bill of rights for scientists and engineers.

## Preamble

A scientist or engineer (S/E) uses understanding, insight, and ingenuity to add to existing knowledge and to create new technologies that benefit both individuals and societies at large. In pursuing these goals, an S/E must be free to theorize and experiment, unimpeded by political pressures, religious dogma, or fear of reprisal. Preservation of this freedom is the purpose of the Bill of Rights for Scientists and Engineers. The Bill of Rights is consistent with the Statement on the Universality of Science of the International Council for Science (ICSU).

## Articles

- An S/E is an individual who uses a logical and systematic approach in the pursuit of new knowledge and technologies. An S/E is not required to possess any specific credential such as an academic degree, appointment in an institution, funding by an agency, or membership in an organization.
- Science and engineering may be practised in any location; they are not confined to academic institutions, government facilities, or industrial settings. An individual using a scientific approach in a home laboratory is pursuing science or engineering as is an S/E employed by an institution, industry, or government agency. No prejudice towards the work of an S/E shall be exercised based on an individual's affiliation or lack thereof, with a particular institution, organization, or agency.
- An S/E shall not be dissuaded from pursuing scientific inquiry because of political or religious concerns, or because the inquiry deviates from a conventional perspective.
- An S/E shall be able to use any approach to new knowledge and technologies, limited only by the restrictions that the approach follows sound scientific principles and does not violate societal ethical precepts.
- An S/E shall be free to collaborate with other individuals in the same or other locations, with the understanding that collaboration may require covenants protecting confidentiality and intellectual property.
- An S/E shall not be subject to restraints in the presentation and publication of results that are imposed by political or religious entities or because the findings conflict with traditional knowledge. Scientific and engineering results should always be evaluated on their merits and not because of preconceived notions of “truth.”
- An S/E shall decide who should co-author scientific publications based on well-established guidelines for co-authorship. Courtesy authorship to senior personnel in an S/E's laboratory or institution is unacceptable.
- An S/E shall strive to ensure that scientific results are widely accessible to the scientific community.
- An S/E should object to the misuse of research findings for political, ideological, or financial purposes.
- An S/E shall always adhere to universal ethical and moral standards.

